# Stigmatization and discrimination of female tuberculosis patients in Kyrgyzstan – a phenomenological study

**DOI:** 10.1186/s12939-025-02566-4

**Published:** 2025-07-01

**Authors:** Rhea Brüggemann, Fabian Schlumberger, Firuza Chinshailo, Matthew Willis, Abdullaat Kadyrov, Gulmira Kalmambetova, Mo Chen, Sophie CW. Unterkircher, Nestan Moidunova, Altynai Sydykova, Anil Fastenau

**Affiliations:** 1 Department of Health Ethics and Society, Faculty of Health, Medicine and Life Sciences, Maastricht University, Maastricht, 6211 LK The Netherlands; 2 National Tuberculosis Program Kyrgyzstan (NTP), Akhunbayeva Street 90a, Bishkek, 720020 Kyrgyzstan; 3 Marie Adelaide Leprosy Center (MALC), Karachi, 74400 Pakistan; 4 Department of Global Health, Institute of Public Health and Nursing Research, University of Bremen, Bremen, 28359 Germany; 5 German Leprosy and Tuberculosis Relief Association (GLRA/DAHW), 97080 Wurzburg, Germany

**Keywords:** Stigmatization, Discrimination, Women, Kyrgyzstan, TB

## Abstract

**Introduction:**

The Republic of Kyrgyzstan is among the 30 countries with the highest burden of multidrug-resistant Tuberculosis worldwide. One of the reasons is widespread stigmatization and discrimination. As previous research has shown, particularly women experience stigma while its impact on their life and (mental) health is even greater than for men. This is the first phenomenological study to explore women’s lived experiences of TB-related stigmatization in Kyrgyzstan. This study aims to raise awareness about the gender-specific impact of stigmatization and discrimination.

**Methodology:**

Descriptive phenomenology was used. 15 semi-structured in-depth interviews with female TB-patients were conducted between 28th May and 14th June 2024. Themes were stigma experiences, their consequences and coping strategies. Participants were recruited from two TB Hospitals and two Family Medical Centers (primary health care units) in Bishkek through purposive sampling. The data analysis followed a thematic approach based on a combination of deductive and inductive coding.

**Results:**

14 of 15 participants experienced stigmatization and discrimination in one way or another. Anticipated stigma was very prominent, manifesting in non-disclosure of the diagnosis apart from close family. Enacted stigma mostly occurred within society or non-TB-specialized healthcare facilities. Self-stigmatization often followed anticipated and enacted stigma. Stigma experiences impacted daily and social life, marital prospects and access to educational and work opportunities but mainly led to mental health issues, which 12 of 15 participants reported.

**Discussion:**

and Conclusion.

In contrast to previous research, this study did not find diagnostic delay nor non-adherence to treatment because of stigmatization and discrimination. However, experiences within the healthcare facilities impacted the perceived quality of care. Stigmatization within the family, mostly by in-laws, was anchored in the patriarchal and conservative attitudes of Kyrgyz society. Overall, key findings of this study were widespread lack of knowledge about the disease and its transmission as a reason for and mental health issues because of stigmatization and discrimination. The findings imply the need for intervention strategies and policies focusing on education about TB, integration of psychosocial support into treatment and improvements in quality of care. Altogether, this could contribute to the reduction of TB-related stigmatization and discrimination which would reduce the individual burden of TB.

**Supplementary Information:**

The online version contains supplementary material available at 10.1186/s12939-025-02566-4.

## Introduction

Despite not only being curable, but preventable, Tuberculosis (TB) returned to being the leading infectious killer worldwide [1]. The Republic of Kyrgyzstan is listed as one of the 30 countries with the highest incidence of multidrug-resistant TB (MDR-TB) worldwide [1]. 39% of the estimated TB incidence rate of 112/100,000 are women [1]. TB is among the top ten leading causes of death in Kyrgyz women between 15–49 [2]. The country’s treatment coverage only amounts to 53% [1] while the treatment success rate of 83% [1] is lower than the WHO target of at least 90% [3]. Low detection rates, inadequate prevention and control measures as well as gender discrimination and human rights related barriers to TB services contribute to the high burden of TB in Kyrgyzstan [4–7]. Another crucial factor is widespread stigmatization and discrimination, which are part of cultural and structural norms [4].

Stigma, “an attribute that is deeply discrediting” [8], can take various forms: anticipated stigma is the fear or expectation of being stigmatized after disclosing the TB diagnosis [9]; enacted stigma refers to actual experiences of differential treatment by others because of the diagnosis [9]; and internalized or self-stigma occurs when individuals apply negative stereotypes to themselves [10]. The process of stigmatization contains six components that occur concurrently: labeling, stereotyping, separation, status loss and discrimination within the context of power asymmetries [11]. Therefore, discrimination describes the actions, behaviors and attitudes following the act of stigmatization [8].

A study by Huffmann et al. [4] showed that among other aspects of quality TB services, quantified stigma levels in Kyrgyzstan such as perceptions of stigmatization and discrimination are higher in urban areas [4]. TB patients experience the highest levels of stigma within the local community, with self-stigma subsequently following. In Bishkek specifically, self-stigma was the most common [4]. Patients further face stigmatization and discrimination from healthcare workers and family members [7]. A study by Burtscher et al. [6], focusing on perceptions and opinions about TB among people affected, found that vulnerable groups experiencing stigmatization are migrants, homeless people, ethnic minorities or women, especially young women. According to the study, a woman suffering from TB “loses her ‘significance’ and is considered inappropriate for the role of a daughter-in-law” [6]. The fear of rejection, getting divorced or not finding a husband is a reason why many young women do not undergo treatment although basic diagnostics and TB treatment in Kyrgyzstan are free of cost [6].

Stigmatization is often caused by public health practices and the public discourse about TB combined with attitudes of healthcare workers towards TB patients [12]. Some studies imply that this stigmatization develops from a lack of knowledge about TB as well as the fear of infection [13–15]. People affected by TB are judged, blamed and shamed for being infected [12]. The public commonly associates TB with poverty, a low caste or assumes that it is a curse or form of punishment for earlier immoral behavior, like alcoholism, smoking or utilizing the services of sex workers [13]. As a result, TB patients often experience self-stigmatization [12]. Drivers for healthcare workers stigmatizing TB-patients are fear of infection, stress from providing care or fear of social and career implications if they became ill. These factors are facilitated by cultural norms, workplace safety standards including a lack of infection control measures and health policies [16].

Various studies examined the impact of health-related stigmatization and discrimination on an individual, displaying psychological, social and economic effects, which altogether impact the quality of life [17–19]. Several studies mention negative effects on the TB patient’s mental health, including low self-esteem, depression, suicidality and other psychological issues [20–22]. Psychosocial consequences include social anxiety, performance and social avoidance as well as isolation and social exclusion [23]. Economic consequences result from loss of employment, fewer work opportunities or reduced income [12, 21, 24]. Although, globally as well as in Kyrgyzstan, more men than women are affected by TB [1, 5], women face more stigma [25]. Additionally, literature investigating gender-related differences in TB stigmatization and discrimination has shown that all these consequences are even more severe for women, complemented by negatively influenced marriage prospects as seen in studies from Bangladesh, Nepal, Pakistan and India [21, 24–28]. A study from Ghana showed that female TB patients experience greater economic challenges, as they are more likely to lose their employment [29]. Additionally, a number of studies portray stigma experiences, particularly within healthcare facilities, or the fear of stigma as a barrier to early diagnosis, adequate care and treatment adherence [14, 25, 29, 30]. Out of fear of stigmatization women hide their diagnosis moreoften and delay their healthcare-seeking, which increases the risk of serious complications, MDR-TB and further transmission of the disease [21, 25].

Stigmatization plays a pivotal role, not only in detecting and controlling, but also in ending TB [24]. To reduce barriers to TB care, stigma research followed by intervention strategies is needed. These stigma interventions as well as TB policies need to include the gender aspect to increase women’s access to quality TB care and to decrease TB-related stigmatization [20, 31].

Since globally, as well as in Kyrgyzstan there is a gap in qualitative research specifically focusing on the experiences of TB-related stigmatization and discrimination and its gender-specific impact, this study adds in-depth insights into women’s lived experiences of stigmatization and discrimination as well as its consequences in Kyrgyzstan. The aim of this study is not only to raise awareness, but consciousness about the impact of stigmatization on, especially, female TB patients. The findings of this study can be of use for the National Program – TB VI (2023–2026) developed by the National Tuberculosis Program (NTP) of Kyrgyzstan. This program focuses on integrated, people-centered care by addressing aspects like the reduction of stigmatization and discrimination combined with social and behavioral change and social support for TB patients [4]. Therefore, the results of this study can be used to inform gender-sensitive intervention strategies and policies surrounding TB stigmatization in Kyrgyzstan in order to decrease the individual burden of stigma within an inclusive, non-stigmatizing approach to TB care [32].

## Methodology

### Study design

This study employed a qualitative research design using descriptive phenomenology to explore participants’ lived experiences of TB-related stigmatization in the social environment and in the medical field [33]. Themes of the interviews were stigma experiences and their consequences as well as coping strategies.

The study was conducted in collaboration with NTP Kyrgyzstan and the German Leprosy and Tuberculosis Relief Association (DAHW). The data collection took place from 28th May to 14th June 2024 in Bishkek, the capital of Kyrgyzstan, where, next to various Family Medical Centers, two Tuberculosis hospitals, the National Center of Phthisiology under the Ministry of Health of the Kyrgyz Republic and the Bishkek City TB Hospital, are located. However, collecting data only in these healthcare facilities limits the generalizability to urban Bishkek.

## Research population and sample

To increase representativeness, the research sample included women with different durations of disease, ethnicities and/or religions, occupations and marital statuses. The participants were between 19 and 59 years, diagnosed with various forms of TB (Table 1). All participants have been diagnosed with and treated for TB for the first time, thus had no previous personal experiences with this disease nor TB-related stigmatization. The following inclusion criteria needed to be met for participants to take part in the study: of legal age, residing in Kyrgyzstan, currently undergoing TB treatment, willingness to participate and complete the written informed consent form along with the ability of expression in Russian, Kyrgyz or English. To avoid ambiguous findings TB patients coinfected with HIV/AIDS were excluded from this study since HIV/AIDS is a disease which is highly stigmatized itself.

**Table 1:** Sociodemographics

	**Age**	**Religion**	**Ethnicity**	**Marital status**	**Employment**	**TB diagnosis**	**Date of Diagnosis**	**Beginning of treatment**
P1	56	Islam	Kyrgyz	Widowed	Not working	Unknown**	End of 02/2024	06/03/2024
P2	48	Islam	Kyrgyz	Married	Not working	Unknown**	16/05/2024	17/05/2024
P3	23	Islam	Kyrgyz	Married	Not working	Lung TB	05/2024	05/2024
P4	21	Islam	Kyrgyz	Unmarried	Student	Bone TB	End of 11/2023	11/2023
P5	59	Christian	Russian	Unmarried	Worked before*	DR-TB	Beginning of 05/2024	26/05/2024
P6	54	Islam	Kyrgyz	Married	Worked before*	DR-TB	11/2023	20/11/2023
P7	25	Islam	Kyrgyz	Married	Worked before*	DR-sensitive TB	End of 05/2024	End of 05/2024
P8	20	Islam	Kyrgyz	Married	Student	MDR-TB	31/01/2024	03/02/2024
P9	43	Christian	Kyrgyz	Married	Housewife	Lymph TB	12/2023	12/2023
P10	59	Islam	Kyrgyz	Married	Working	Unknown**	06/2023	06/06/2023
P11	29	Islam	Kyrgyz	Divorced	Worked before*	Lymph TB	Unknown***	23/01/2023
P12	31	Atheist	Kyrgyz	Married	Maternity leave	MDR-TB	06/2023	06/2023
P13	19	Islam	Kyrgyz	Married	Student	Closed TB	Beginning of 06/2024	03/06/2024
P14	26	Islam	Dungan	Married	Not working	Lung TB	05/2024	14/05/2024
P15	27	Islam	Kazakh	Married	Working	Lung TB	04/2024	04/2024

*DR-TB* form of TB that is resistant to at least one first line anti-TB drug, *MDR-TB* form of TB that is resistant to both isoniazid and rifampicin (most potent anti-TB drugs)

^*^ termination of work is not directly related to TB diagnosis

^**^ participant did not know their exact TB diagnosis

^***^ inconsistent data

After obtaining ethical clearance, NTP Kyrgyzstan connected the researcher with facility heads and healthcare personnel, who identified potential participants through purposive sampling. Healthcare workers provided information sheets to female, non-infectious TB patients undergoing treatment. Interested patients informed the staff, who then introduced the researcher and translator. Following clarification of questions and obtaining informed consent, interviews were conducted at the healthcare facility. Initially participants were recruited as inpatients from two TB Hospitals in Bishkek. Due to the refusal of participation of all potential participants at the National Center of Phthisiology, the recruitment strategy was advanced to recruiting outpatients from two Family Medical Centers in Bishkek to achieve the initial target of 12 to 15 participants, which was expected to achieve data saturation while prioritizing a rich and detailed understanding of participants despite time constraints.

### Data collection procedures and measures

Fifteen in-depth, face-to-face interviews lasting 20–90 min were conducted by RB and FC in a privacy ensuring setting. Topics were drawn from prior research on health-related stigmatization and discrimination [17, 21]. The semi-structured interviews followed a guiding set of standardized, open-ended questions, reviewed and revised by researchers RB, FC, MC, AF and FS before and after initial interviews to incorporate new insights. The gender-matched researcher RB recorded the interviews and took notes to capture non-verbal cues. Before the interviews, participants completed a sociodemographic questionnaire, including age, religion, ethnicity, marital status, employment, TB type, diagnosis date, and treatment start date.

During the interviews, FC, a NTP staff member, fluent in source (Russian/Kyrgyz) and target (English) languages and familiar with the cultural and contextual nuances of the topic served as a translator. To facilitate communication, translations were primarily non-simultaneous, and in some instances participant statements were summarized rather than literally reproduced. While the transcript not always reflects the participants’ exact wording, the translator was briefed to prioritize meaning and preserve the intent and tone of responses. When necessary, clarification was sought during the interviews to confirm intended meanings. To ensure reliability in translated quotes, a glossary of key thematic terms (e.g. stigma, discrimination) was used to support consistency in translation within and across interviews.

### Data analysis

The recordings were transcribed verbatim and initially analyzed by RB using a thematic approach [34] with the support of ATLAS.ti 24 software. A coding guide was developed to ensure validity and consistency. This coding guide included deductive codes, themes, and categories adapted from prior literature. While reviewing the transcripts carefully, these deductive codes were refined and expanded through inductive codes which were newly developed during the analysis. The combination of deductive and inductive coding was used to identify patterns. The initial coding was reviewed and triangulated by MC, AF and FS based on the coding guide. Discrepancies in coding were discussed in detail and resolved through consensus to informally achieve inter-coder agreement. Therefore, comparing interpretations resulted in a refined coding framework. Beginning with one interview and repeated across all transcripts, this process continued until no new codes, categories or themes emerged, indicating data saturation at fifteen interviews.

Results are illustrated by quotes to highlight typical findings or variations. To enhance readability, quotes were rewritten to the first person where necessary. Filling words and stutters have been omitted while severe grammatical errors have been corrected.

### Ethical considerations

Local ethical clearance was granted by the Ministry of Health, Kyrgyzstan (01–8/14). Additionally, this study obtained ethical approval from the FHML-REC of Maastricht University (FHML/GH_2024.022). Participation in this study was completely voluntary, informed consent was obtained prior to the interviews. Participants had the right to withdraw from the study at any point of time without providing a reason. All interviews were recorded, transcribed and stored without any personal information to guarantee data privacy and confidentiality.

## Results

From the data, four main themes were identified: 1) experiencing TB, 2) stigmatization and discrimination experiences, 3) consequences and impact of stigmatization and discrimination experiences and 4) coping.

### Theme 1: Experiencing TB

#### Reactions to the Diagnosis and Consequences of TB

Participants showed different, even opposite reactions to their diagnosis, such as relief or feelings of shock and panic. Participants who were mothers were mostly worried about infecting their children. Others were afraid of the disease, the treatment or the reaction of other members of society. All these feelings affected the participants’ mental health. However, learning that TB is a curable disease with free treatment for everyone helped the interviewed women to accept their diagnosis.

Moreover, TB was found to have a significant impact on a person’s life. Patients were restricted in their daily activities due to their type of TB and the following treatment regime. The treatment of TB was associated with various side effects of drugs which adversely impacted the overall health condition, mental health and quality of life of patients. They also presented as a reason to discontinue treatment.

#### Knowledge and Perceptions about TB

The interviews showed a widespread lack of knowledge about TB within Kyrgyz society. People did not know about different forms apart from lung TB, nor that those forms are typically not infectious. This manifests in the predominant fear of infection. Moreover, people believe that TB is not treatable or, on the contrary, treatable with pseudo medicine, such as alcohol or “*dog soups*” and “*drinks from the snakes*” (P8) as forms of traditional medicine. Almost all patients mentioned that they themselves did not know anything specific about TB prior to receiving their diagnosis apart from being a highly infectious disease with a challenging treatment.

The lack of knowledge translates into public perceptions about TB. The participants mentioned some prevailing myths and misbeliefs. For example, some people had the “*perception that if you are pregnant with TB, you cannot bear a healthy child”* (P13) although TB cannot be transmitted from mother to fetus [35]. However, one of the main beliefs about TB described the “kind of people” that are infected:

*“I thought that TB was diagnosed only for those who have no appropriate work, like sex workers or in prisons. I thought it was only this kind of people, the low attitude people, but I saw there were normal people that have work and other things in the hospital.” (P5)*

*“I heard that mostly TB is diagnosed in alcoholic people, homeless people and also drug addicted people (…).” (P7)*

These beliefs were widespread among the patients as well as the community. Further, some people, including healthcare workers, thought that TB is a “social disease” which depends on the living conditions or the nutrition. Consequently, TB patients not only have to fight the disease, but also other people’s perceptions which might lead to stigmatization and discrimination towards TB patients.

### Theme 2: stigmatization and discrimination experiences

Most women interviewed (14/15) reported some form of stigma experiences, either their personal ones, or the ones they have seen or heard of. These experiences can be categorized as enacted, anticipated and internalized stigma.

#### Enacted stigma

The participants described experiences with stigmatization and discrimination in different settings such as family (parents, siblings, husband, children) and relatives (grandparents, aunts, uncles, cousins, family-in-law), friends, at work and in university or from the public. These experiences include situations where the participants either felt they were treated differently because of their TB status, noticed changes in the interaction or received negative reactions after disclosing their diagnosis. Sometimes, they even classified it as stigmatization or discrimination themselves.

1. Family and relatives

Most interviewees stated that their close family did not show negative reactions after disclosing their diagnosis but feared infection. The participants did not identify changes in the relationship or everyday life. Most interviewees perceived the family as very supportive. However, participants highlighted negative reactions and avoidance by extended family, primarily from the husband’s relatives:

*“My own relatives from my side, they understand everything, and they visit me but relatives from the husband's side they don't want to visit me, they don’t speak with me and support the conversation, the bond, relative bonds.” (P6)*

Concerns about her ability to care for children or the household or to bear healthy offspring also led some in-laws to deny her in-patient treatment, despite her own preference.

One participant reported verbal abuse from her mother-in-law who feared her son could get infected. Relatives from her husband’s side blamed her for potentially infecting her young child. They demanded she leaves for the hospital and does not return until the treatment was complete. This participant shared that her TB diagnosis led her to initiate a divorce, as she felt stigmatized by her husband at times:

“*Sometimes when I argue with him, he starts to say ‘I need to divorce you’ because he’s going to get infected. My husband said (…) ‘you need to be isolated from the community’, this kind of things.”*

Financial dependency and verbal abuse further influenced her decision:

“*I started to financially depend on him. I was on maternity leave. I have this disease, and I cannot work. And he started to abuse me because of this. And it let to this divorce process”. (P12)*

Another participant recounted similar behavior of her in-laws:

*“They don't want me to be their bride (daughter-in-law). My family members started to abandon me. They were very shocked, and they even tried to abandon me”. (P13)*

Her husband’s family avoided her out of fear of infection. The mother-in-law never visited after her diagnosis. However, in this case, her husband provided consistent support despite his family’s actions.

(2) Friends and acquaintances

In case participants decided to tell their friends, they received positive reactions. Close friends were very supportive, they came to visit and kept the participants updated about things going on outside of the hospital. Other participants did not tell their friends because they were afraid that they would not talk to them anymore or simply based on the belief that their health condition is their personal business. Often non-disclosure of the diagnosis goes hand in hand with self-stigmatization when participants blamed themselves for others needing to pass examinations to rule out an infection. In addition to that, they were ashamed of their diagnosis resulting in self-stigmatization. Consequently, they isolated themselves from their friends as shown drastically in a statement of participant 13:

*“I don't say about my diagnosis to my university mates. But they know. (…) they all have to pass this x-ray examination (…) because of me. I don't want to come back to my own city. I don't want to see them (…) I changed my phone number; nobody can call me because it's a very small city and anyone can then know about this. I'm afraid. I don't want to talk with them because I think that they will underestimate me, make this my low self-esteem.” (P13)*

(3) Healthcare workers

Overall, the participants were very content with the treatment in the TB hospital, speaking highly about their nurses and doctors, who not only provided quality treatment, but also were friendly and supportive. Negative experiences within the healthcare sector either occurred at the facility where the diagnosis was given (primary healthcare level) or at non-TB healthcare facilities. Several participants mentioned they did not like the way their diagnosis was delivered. They wished healthcare workers would prepare them better for receiving this diagnosis, provide them with more information about the disease and deliver the diagnosis in a kinder way instead of telling it “*with contempt” (P1).* Several participants experienced a change of behavior in healthcare staff once those found out about their TB diagnosis, perceiving them as rude and applying excessive precautions. This made them feel of less worth and not equal to other patients.

(4) Society

Some participants directly experienced stigma within society, which affected their daily life. One participant recounted losing her apartment due to her and her son’s TB diagnoses:

*"The apartment owner got to know this and just kicked us out without any delaying of the stay."* (P6).

Unable to secure new housing immediately, their belongings were left on the street. This experience had a deep emotional impact, leaving the participant afraid of a similar situation with the new landlord.

Another instance of stigma involved taxi drivers. Upon realizing the destination was the TB hospital, the driver grew visibly anxious and wore a mask, despite reassurances from the women that they were not infectious. This reaction left the participants feeling uncomfortable and judged.

#### Anticipated stigma

Overall, anticipated stigma seems to play a big role. Apart from telling family, relatives and sometimes the closest friends, the participants usually did not tell anyone else voluntarily about their disease. They did not share their diagnosis at work or with neighbors. Even if some participants said they do not have stigmatization experiences, often, they anticipated stigma from certain groups or were afraid of public perceptions. They were afraid of negative reactions or that people’s attitudes towards them would change, they worried that *“others will treat (me) differently if they will know about this disease, even they will avoid me in the street” (P6).*

#### Internalized Stigma

Several participants expressed self-stigma as a response to their TB diagnosis, either directly or as a reaction to others' attitudes. They described feelings of shame, humiliation, and self-blame:

*"I have my own battle. I start to blame myself,'Why do I have this treatment?'(...) It's a shame to tell them. (...) I started to isolate."* (P11)

As a result, participants isolated themselves and avoided others to prevent infection even when they were no longer infectious. They tried to prevent any possibility of blame, self-imposed or from others, for spreading the disease. Some struggled to accept their diagnosis, questioning why they, out of all people, had been infected. Occasionally, a participant explicitly acknowledged experiencing self-stigmatization.

#### Observed Stigma

In addition to their own experiences, many participants had seen or heard of others being stigmatized due to their TB status. They witnessed these situations directly or learned about it in conversations with other patients at the healthcare facilities:

*“I heard a lot of (stories) when there are people with TB who were abandoned from the family, that suffer very much because of this disease and because of the reaction of their family.” (P8)*

They witnessed other female patients being blamed for getting infected, being separated from their children or not supported in any way, neither financially nor emotionally.

### Theme 3: Consequences and Impact of stigmatization and discrimination experiences

*“I feel that my life is divided in two parts, before the treatment and after the treatment, before TB and after TB”* (P8)—Coming with the diagnosis, stigmatization and discrimination experiences lead to various consequences on different parts of a TB patients’ life.

#### Daily and social life

Consequences of the stigmatization and discrimination found in this study were the (temporary) exclusion from university classes or work opportunities:

“I *was afraid, I was sad that there is no way to study if you have TB during your treatment and also you cannot work during your treatment. I was called from the university, and they said, ‘either you will quit the university, or you will have this gap year’” (P8).*

This exclusion not only denied access to education and employment but also came with economic insecurity or dependency on the husband or family.

Participants often concealed their diagnosis from the public, driven by anticipated stigma. Observing others being stigmatized increased their reluctance to disclose their condition, fearing similar reactions or avoidance by family members. This fear, coupled with concerns about infecting others, led many to isolate themselves, avoiding social interactions. Some participants reported losing contact with friends and family due to their TB diagnosis. Additionally, marital prospects were adversely affected. In one case stigmatization led to, at least, temporary homelessness.

Stigmatization and discrimination have an impact on the quality of healthcare. As a reaction to the stigmatization they experienced from healthcare workers, some patients looked for different ways of receiving healthcare by changing the facility. One patient said she interrupted her treatment and ran out of the hospital because of “*the attitude of the medical doctors” (P13).* Then she changed to another one.

#### Mental health

The psychological impact of a TB diagnosis was profound, with stigmatization and discrimination significantly affecting participants' emotional well-being. Many described feeling deeply uncomfortable in situations where they were treated differently, leading to stress and worry when reflecting on those experiences. For some, the experienced situations diminished their self-esteem, making them feel devalued because of their TB diagnosis.

Twelve of the 15 participants reported mental health effects stemming from stigma, ranging from general distress to depression and suicidality. In some cases, the diagnosis, combined with anticipated stigma, triggered thoughts of death:

*"When they said it’s TB, I didn’t want to live, the community doesn’t want to accept me. It was very difficult for me when I heard."* (P10)

Such thoughts also emerged as a consequence of stigmatization experiences. Some participants witnessed others struggling with their mental health due to stigma experiences, increasing their own emotional burden and potentially impacting their treatment as stated by Participant 15:

*"Recently, I had a small conversation with another woman here. She said to me, ‘I don't want to live anymore… I want to commit suicide. Tomorrow, either I will leave this hospital, or my body will be taken out.’"* (P15)

### Theme 4: coping

To handle situations where they were stigmatized, participants named various coping mechanisms that help them deal with induced stress or mental health issues. These strategies are also used to deal with the disease itself and its consequences.

#### Support

*“It’s all about support of my family.” (P7)—*The key coping mechanism identified was (mental) support, particularly from family and spouses. Most participants (12/15) reported having supportive families that helped them manage the disease and stigmatization experiences, with some highlighting the significant impact of their husbands'support. Healthcare facilities served as an important source of support, primarily from fellow patients, as well as healthcare workers and doctors:

*“The support of the doctor and my family returned me to my daily life, to the way that I was before I was diagnosed.” (P2)*

Support from friends, classmates, or colleagues also played an important role in enabling patients to maintain their daily lives during and after treatment. Regular check-ins, calls, and visits were particularly valuable in facilitating the treatment process.

#### Coping strategies

Other coping mechanisms identified included turning to religion (praying, believing), maintaining economic independence through online work, and engaging in activities like reading books or watching movies to distract themselves from dwelling on negative experiences. A primary focus for participants was adhering to their treatment regimen, ensuring timely medication intake. Those lacking knowledge about TB proactively sought information through healthcare workers, reading, and discussions with other patients.

Sharing experiences with family members, friends or especially fellow patients who faced similar challenges proved impactful. These conversations provided reassurance, normalized their struggles, and offered positive examples of overcoming TB and its social implications. Interactions with other patients also served to challenge public misconceptions about the disease.

Reflecting on positive aspects or comparing their situation to others with greater hardships than TB helped some participants shift their perspective, as one shared:

*“This young lady said that ‘you know that you are the luckiest one at the moment. You know that I was abandoned from my relatives and parents, (…) but you have these supportive relatives and parents’. And after that I just realized this. It became easier for me to get this diagnosis and this situation made a very huge impact for me. “*

Some tried to “*not condemn”* but understand the stigmatizing person. Finding reasons for their behavior in “*saving their health condition”* (P9) helped overcoming negative interactions.

In the hospital, patients supported each other and formed bonds and even friendships through shared experiences. Positive interactions, provided moments of joy and relief from the challenges of TB:

*“For me there are many positive situations, many positive memories from this hospital, from the other patients. (…) We played together, went to the karaoke together, and even celebrated birthdays together.” (P8)*

**Fig. 1:**
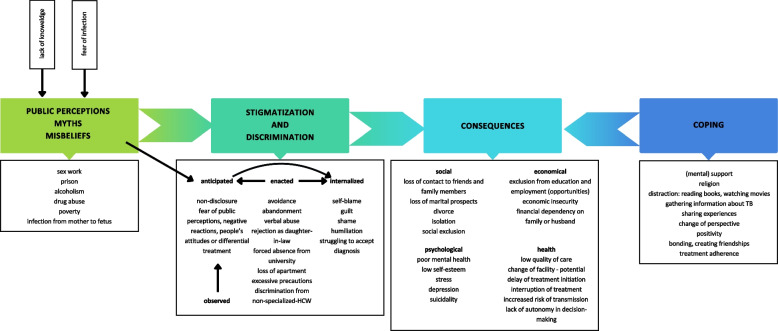
Causes, manifestations and consequences of TB-related stigmatization and discrimination towards female TB patients in Kyrgyzstan

Figure 1 highlights the key findings of this study. It presents the causes of stigmatization and discrimination, its manifestations and consequences which can be counteracted using coping strategies.

## Discussion

This study presents the first analysis of how female TB patients in Bishkek, Kyrgyzstan experience TB-related stigmatization and discrimination and its consequences.

### How do female TB patients in Kyrgyzstan experience stigmatization and discrimination?

Almost all participants of this study encountered stigmatization and discrimination in one way or another although they often did not recognize it as such. Stigmatization can be felt and interpreted differently based on personal, social and cultural factors leading to varied reactions even within the same stigmatized group [36]. Some participants denied experiencing stigma but later described at least one situation where they were treated differently, expected stigma or witnessed others being stigmatized due to TB. This highlights that stigmatization and discrimination present abstract concepts which differ in its perceived severity due to subjectivity [36]. It is a fine line distinguishing between stigmatization and self-protective behaviour aimed at preventing infection. However, from the perspective of those affected, even self-protection may be perceived as stigma. Either of these scenarios can be attributed to a lack of knowledge about TB transmission which is, along with the perceived infectiousness one of the main sources of stigmatization in different countries such as Nepal, India or Ghana [12, 13, 15, 21] as well as Kyrgyzstan, as the interviews confirmed. One might argue that the latter is a consequence of the first. Since the only knowledge patients and society had about TB is its contagiousness and its tough treatment, fear of infection seems a plausible justification for their behavior. Family members becoming more supportive once they received accurate information about TB from healthcare workers or patients themselves underscores the importance of education in fighting stigma and discrimination. Public health campaigns focused on improving knowledge about TB are essential to reduce fear of infection as well as stigma [26]. Additionally, the lack of knowledge is directly aligned with perceptions and beliefs about TB and TB-patients, and the associations people have with other conditions, such as poverty (homelessness) or behavior seen as inacceptable by the society (sex work, substance abuse, prison). Since existing studies found similar beliefs about TB, these ideas seem to be persistent over time and consequently, still lead to stigmatization and discrimination [13, 25, 28].

Experiences within the healthcare sector varied significantly between TB-specialized and non-TB-specialized healthcare workers, as well as between TB hospitals and Family Medical Centers. Stigmatization predominantly originated from non-TB-specialized healthcare workers during treatment for other conditions or at Primary Healthcare facilities during initial, unspecialized care visits. Aligning with findings by Cremers et al. [26] in urban Zambia, participants described non-TB workers in general healthcare centers as rude and criticized the way the diagnosis was delivered. In contrast, participants were satisfied with the care provided in the TB department [26], highlighting disparities in the quality of care between specialized and unspecialized services. Stigmatizing behaviour by healthcare workers, primarily driven by a fear of infection [16], underscores a persistent lack of knowledge even among healthcare professionals—non-TB-specialized—about TB, its various forms, modes of transmission, and infection control guidelines.

If stigma occurred within the family, it was typically from extended family members, particularly the mother-in-law and other relatives on the husband’s side. Rejecting the participant as the daughter-in-law, questioning her ability to manage household duties, care for children or bear healthy offspring is anchored in the patriarchal and traditional attitudes of the Kyrgyz Society, where good health and physical condition are key factors in the selection of a daughter-in-law [6]. In some cases, it seems to be more important to the in-laws that the daughter-in-law fulfills her role than receiving adequate treatment. These conservative attitudes, which appear to be resurging [5], highlight how stigma often is specific to women, shaped by gender inequalities and traditional gender roles. Moreover, these experiences are closely tied to the country’s social context, emphasizing the need to focus on women’s perspectives and the unique consequences stigmatization and discrimination have, increasing the burden TB itself already puts on them.

All in all, stigma experiences of female TB patients in Kyrgyzstan are deeply rooted in cultural expectations and traditional gender roles while health system inadequacies, particularly the lack of knowledge among non-specialized healthcare workers, reinforces the stigma. Thus, addressing stigma requires tackling both knowledge gaps and societal norms.

### What consequences do TB-related stigmatization and discrimination have on a women’s life and their health in Kyrgyzstan?

Unlike previous studies from various countries, mostly Asian or African, [4, 19, 21, 24], this research found no significant delays in healthcare seeking behavior, diagnostics nor low treatment adherence due to stigmatization. On the contrary, participants were determined to complete their treatment, despite facing stigmatization and discrimination from healthcare workers which reduces the quality-of-care patients are receiving. However, often, they were seeking alternative ways of receiving healthcare rather than discontinuing treatment. While finding a new facility might slightly delay treatment initiation, all participants began their treatment promptly after diagnosis. When participants considered interrupting treatment, it was typically linked to severe drug side effects rather than stigma, one of the most common reasons for non-adherence [37]. When delays in seeking healthcare occurred, this was primarily due to lack of awareness about TB symptoms, as most participants did not suspect TB as their diagnosis. While stigma did not directly impact detection or treatment compliance, it can hinder TB prevention and control. Women hide their diagnosis more often than men [25], with almost all participants sharing it only with close family, similar to findings by Mukerji & Turan [21]. Non-disclosure, primarily driven by anticipated stigma, combined with lack of knowledge, increases the risk of transmission due to inadequate precautions [22]. Consequently, stigmatization and discrimination might secondarily impact the TB prevalence in Kyrgyzstan. Similar to other research from Zambia and South Africa [20, 26], in this study anticipated stigma appeared to play a major role. As a consequence, TB patients avoided others and isolated themselves, which are typical symptoms of internalized stigma [21]. Consequently, self-stigma can not only be part of the stigma experiences but also be a consequence of other forms of stigma TB-patients have encountered.

Liamputtong & Rice [36] describe that “the consequence of stigma is that the stigmatized are systematically excluded from life chances and opportunities such as education, housing, employment, and health and social care” which has also been the case in this study. Here, stigma impacts marriage and the quality of care. It further affects the daily and social life and can have severe economic consequences through exclusion from work or university. Altogether these negative consequences on various domains of an individual’s life increase the individual burden of the disease. But most of all, stigma experiences have an impact on the patient’s feelings. Often, they lead to mental health issues of varying intensity, ranging from feeling uncomfortable, over depression up to suicidality similar to the findings of Mukerji & Turan [21]. Stigmatization from healthcare workers especially impacts the self-esteem of the patients. TB-patients experiencing internalized stigma were found to be more likely to develop depression [22]. Altogether, psychological issues present the most severe complications of TB itself as well as TB-related stigmatization and discrimination. The mental health effects often stem not only from the direct impact of stigmatization and discrimination but also from secondary consequences, such as homelessness, the loss of friendships or family connections, or divorce.

### How do female TB patients cope with stigmatization and discrimination?

Overall, the coping mechanisms found in this research align with those found by Mukerji & Turan [21] who divided the strategies into positive and negative coping. Under negative coping they mentioned self-imposed isolation. However, in this study isolation as part of self-stigmatization is seen as a consequence of stigmatization experiences, especially anticipated stigma, rather than a coping strategy. Although they felt uncomfortable in situations where they were treated differently because of their diagnosis, participants themselves often tried to change their perspective to make sense of someone’s behavior. A study conducted in Ghana showed that a change of perspective could give insights into other’s fears [38]. Thus, participants could empathize with people who treated them differently. Understanding that there is no malicious intent behind the stigmatization helps to cope better [38]. Next to common strategies such as turning to religion, distracting oneself through music or television, the findings of this study, as well as from Mukerji & Turan [21] show that support from family, healthcare workers and friends is crucial to overcome TB and related stigmatization. A predominant strategy mentioned by the participants is grouping up with other patients; sharing experiences, feelings and information, supporting each other and creating friendships.

None of the participants mentioned the use of psychosocial support as a coping strategy. This could indicate nonexistence of adequate offers for counselling, psychological and social support. Huffmann et al. [4] determined that all over Kyrgyzstan only a third of healthcare facilities offer TB support groups while three-quarters of patients received counselling. This counselling is mostly provided by doctors or nurses, not by trained counsellors. In addition, psychosocial support is less available at the tertiary healthcare level [4]. Another reason why participants did not seek professional help, also outside their TB facilities, could be stigma and discrimination of people with mental health issues and even their family members [39]. This shows that in Kyrgyzstan there is a lack of availability of mental health services combined with stigma and discrimination of mental disorders which further reduces individuals’ initiative to seek for help. This might explain the absence of participants mentioning the use of psychosocial support as a coping strategy.

### Practical implications and recommendations

Stigmatization and discrimination interventions should include all dimensions; anticipated, experienced and internalized stigma. Translating the findings into practice, several implications drawn from this study can be used to inform the “active government policy on TB control, social support, stigma and discrimination reduction” [40] aimed for in the Tuberculosis VI Program (2023–2026). Three primary areas of focus have been identified:

1. Public Awareness Campaigns:

To improve knowledge about TB, public health campaigns that distribute information about the disease, its different types, symptoms and transmission should be initiated as part of stigma reduction programs. These campaigns could take place in schools and universities, healthcare facilities or through print and online media to reach family, friends and the community of TB-patients as well as healthcare workers and the broader society. The primary aim is to raise awareness for the preventability and curability of TB [6]. By raising awareness about the etiology of the disease, existing misbeliefs, myths and perceptions about TB can be deconstructed, which again could prevent stigmatization and discrimination against TB-patients. Additionally, the gender aspect needs to be emphasized, as shown in this study. The content of the campaigns needs to be easily understandable without prior knowledge, so that societal attitudes can be changed by improving education [26]. Since TB is a widespread disease in Kyrgyzstan, raising awareness is of national interest. The proposed awareness campaigns will be a helping factor in reaching Kyrgyzstan’s TB reduction goal of 90% by 2030 and can be funded via NTP Kyrgyzstan.

(2) Upscaling of Psychosocial Support:

The lack of psychological support within the hospital could be improved by implementing a two-fold strategy based on former TB patients and professional psychologists. Support groups consisting of current and former TB-patients build the first stage, enabling patients to share their experiences and feelings, exchange coping strategies and support each other. As seen in the findings of this study, creating supportive environments and fostering a sense of belonging significantly contributes to the well-being of TB patients. The groups could be led by former TB-patients, allowing patients to access first-hand information and to have a role model. Additionally, a mentor system can be adopted to provide one-on-one peer counselling, if required. As the second pillar of this strategy, trained psychologists should be available to provide professional assistance in severe cases like suicidal thoughts participants in this study mentioned. Having both professional psychologists and volunteers (former TB-patients) available for psychological support can play a significant role in dealing with (self-)stigmatization. Having the former patients as volunteers at the center of the strategy is not only cost-effective, but also lowers the barrier to seeking psychological help. To ensure the early implementation of psychosocial support in treatment, TB-patients should also be screened for mental health issues and internalized stigma at the time of admittance into the hospital. For this, questionnaires and self-stigmatization scales can be used. These psychosocial interventions need to make sure to account for gender differences in needs, experiences and access. This two-fold strategy could firstly be implemented at the TB hospitals and later on be expanded and adapted to work on a larger scale across all healthcare levels.

(3) Improvements in Healthcare:

To ensure that TB-patients “receive the care they deserve” [4], the quality of care needs to be improved by reducing TB-related stigmatization within the healthcare system. To improve the situation, especially in primary, non-TB specific facilities, policies to promote TB sensitivity amongst healthcare staff should be implemented*.* Healthcare staff needs to be educated on different types of TB, their infectiousness as well as existing infection control guidelines to apply adequate but not excessive precautions. This can be achieved by adding TB sensitivity trainings to the general curriculum in healthcare staff education and by offering in-house trainings for existing staff. These trainings also need to emphasize communication skills on how to respectfully deliver the diagnosis. Through this double-sided approach, both existing and future staff will be able to provide better care for their patients before passing them on to TB-specialized facilities or when treating other medical co-conditions. Improvements in the quality of care contribute to a more patient-centered approach of care in the Kyrgyz healthcare system as it is part of the Tuberculosis VI Program.

## Strengths, limitations and future research

The qualitative research design allows in-depth insights into the stigmatization and discrimination experiences of female TB-patients. This study does not give an overview of the overall situation of stigma within Kyrgyzstan but reflects on individual experiences and the impact they have on a female TB-patients life. By specifically investigating only women’s experiences a marginalized and under-researched population has been given special attention. It portrays their experiences in detail, so that intervention strategies can be tailored specifically to women’s needs. However, although planned, only focusing on female TB-patients does not explicitly show which stigma is gender-based nor that women are more affected since no comparison to men’s experiences has been made. In future, a comparison study between male and female TB patients could be made to not only quantify who experiences higher levels of stigma and to show the differences in the burden of stigmatization but also to understand how men experience TB-related stigmatization and discrimination and their consequences. Including the male perspective in future research will help shaping inclusive stigma-reduction strategies by focusing on gender-specific needs.

Stigmatization and discrimination are very sensitive topics many TB patients might not want to talk about. Due to self-selection bias and the voluntary nature of participation in this study, it must be assumed that those who chose to participate might not represent the entire target population. TB-patients who are highly stigmatized may not want to participate and share their stories. This means that those who are choosing not to be part of this research differ from those who do in aspects that are critical to the outcome of the study. The nonresponse bias was particularly present in one of the TB hospitals, where all approached female TB-patients refused to participate based on unknown reasons. Additionally, there has been a potential social desirability bias due to the presence of an NTP staff member as translator which might have influenced participants’ openness to share sensitive information. However, beforehand it has been repeatedly stated that this interview will not affect the participants’ TB treatment and that all responses will be kept confidential. To create an even deeper understanding of the stigmatization and discrimination experiences a mixed methods study could be interesting. For that, stigma assessment scales could be combined with interviews. This would allow fully anonymous responses and provides an opportunity to make use of additional quantitative methodology.

To protect the health of the researchers, only non-infectious patients were interviewed, excluding infectious TB patients who might have experienced higher stigmatization in comparison to patients that have never been infectious. Since Bishkek is the capital and biggest city of Kyrgyzstan, the results found in the study population from Bishkek might not be representable for the whole country due to big differences between the urban and rural areas in Kyrgyzstan. Investigating stigmatization and discrimination experiences in other areas of Kyrgyzstan could show different results, especially between rural and urban areas due to stronger traditional and cultural beliefs or differences in infrastructure.

Due to the small sample size and the influence of societal factors on stigmatization and discrimination, it is unclear whether the findings can be applicable to other countries as well. In general, a larger scale study investigating the impact of stigmatization and discrimination on female TB-patients in Kyrgyzstan could verify the results found in this study. Cross-national studies could point out the contribution of a country’s social context to the burden of stigma.

## Conclusion

The findings of this study show that stigmatization and discrimination continue to be a widespread phenomenon that significantly adds to the already existing burden of TB on an individual by adversely impacting various areas of life. The findings also show that some of the consequences of anticipated, internalized and enacted stigmatization experiences are specific to women. The burden of stigma for female TB-patients is deeply connected with the social context of Kyrgyzstan. Understanding the experiences of female TB patients could contribute to reducing discrimination against women while improving their quality of life and thus, play a part in improving gender equality within Kyrgyzstan. A lack of knowledge as a cause for and mental health issues because of stigmatization present the key findings of this study. The lack of knowledge is consistently observed among patients, healthcare workers or the public. The lessons learnt from this study imply that the focus of intervention strategies and TB policies should lie on the education about TB to reduce the occurrence of stigmatization and discrimination. Additionally, integrating psychosocial support in the treatment which considers gender-sensitivity and scalability could help in reducing the impact of stigma experiences. Therefore, acknowledging individuality in experiences is important. Combined with a higher quality of care the individual burden could be decreased within an integrated, people-centered approach of care as it is the aim of the National Program – TB VI (2022–2026). All these improvements can, secondarily, positively impact the detection, prevention and control of TB in Kyrgyzstan and thus, help to achieve the targets of the WHO’s global End TB Strategy in 2035: “95% reduction in number of TB deaths, 90% reduction in TB incidence rate compared with 2015 and 0% TB affected families facing catastrophic costs due to TB” [3]. All in all, this will contribute to “ensure healthy lives and promote well-being for all” (SDG 3, UN), while it helps to “achieve gender equality and empower all women and girls” (SDG 5, UN) and “reduce inequalities within and among countries” (SDG 10, UN).

## Supplementary Information

Supplementary Material 1.

Supplementary Material 2.

Supplementary Material 3.

Supplementary Material 4.

Supplementary Material 5.

## Data Availability

No datasets were generated or analysed during the current study.

## Abbreviations

DAHW: German Leprosy and Tuberculosis Relief Association
DR-TB: Drug-resistant Tuberculosis
MDR-TB: Multidrug-resistant Tuberculosis
NTP: National Tuberculosis Program
TB: Tuberculosis
WHO: World Health Organization
