# Supplementary material for: Stigmatization and discrimination of female tuberculosis patients in Kyrgyzstan – a phenomenological study
Source: Int J Equity Health. 2025 Jul 1;24:185. doi: 10.1186/s12939-025-02566-4 (PMC12210680; doi:10.1186/s12939-025-02566-4)
Supplement: Supplementary file 1 — Supplementary Material 1. [file 12939_2025_2566_MOESM1_ESM.docx]

**Appendix 1: Definitions shared with the participants**

**Stigma** means labelling someone with an attribute that differentiates and distinguishes that person from others. The person therefore ought to be avoided by other members of society. Stigma is often described as a process of devaluation, whereby a person is discredited, seen as a disgrace, or perceived to have less value or worth in the eyes of others. Some common examples of stigma related to TB include assuming that someone with TB also has HIV, that he/she must be a drug or alcohol user, that he/she is poor or from a low caste and that he/she must have done something bad to deserve the punishment of having TB. This devaluation is then used to justify social isolation and discrimination against the person with TB.

**Discrimination** involves treating someone in a different, unjust, unfair or prejudicial manner, often on the basis of his/her belonging – or perceived belonging – to a particular group. When stigma is acted upon, the result is discrimination. Discrimination consists of actions or omissions that are derived from stigma and directed towards those individuals who are stigmatized. TB-related discrimination occurs when someone is treated differently and to his/her disadvantage. This is because the person is known to have or have had TB or be closely associated with people with TB, such as their spouse or other members of their household. Some TB-related discrimination examples include: not being attended to at hospitals or clinics after TB was diagnosed; being given inferior medical treatment; being kicked out of home by family members upon knowledge of TB diagnosis; being asked not to attend religious services or community events despite being on treatment without any coughing; not being served by neighborhood market vendors; being fired by employers upon knowledge of TB diagnosis; and being refused to return to school despite being on treatment without any coughing.

When we refer to stigma in this interview, we include both stigma and discrimination.
