# Supplementary material for: Stigmatization and discrimination of female tuberculosis patients in Kyrgyzstan – a phenomenological study
Source: Int J Equity Health. 2025 Jul 1;24:185. doi: 10.1186/s12939-025-02566-4 (PMC12210680; doi:10.1186/s12939-025-02566-4)
Supplement: Supplementary file 2 — Supplementary Material 2. [file 12939_2025_2566_MOESM2_ESM.docx]

**Appendix 2: Interview Guide**

**Warming up questions:**

- How are you feeling? Do you enjoy the sun? Have you had a coffee? …
- When did you get your TB diagnosis? What TB diagnosis did you get?

**TB and self-stigma:**

- What are your feelings about TB?
- Have any of these feelings you have about TB inhibited you from seeking and accessing TB services?
- What are your feelings about your TB diagnosis/your TB status?
- How do you feel about yourself?
- What did you think about people with TB before?

**Experiences with others:**

- What are the perceptions/feelings of your family and relatives/friends about TB?
- What are perceptions of your community, colleagues about TB?
- What are public perceptions/beliefs/knowledge you know about?
- How did people react when you told them about your diagnosis? -> How do you feel about this? (Who did you tell/not tell and why?)
- How do you feel about telling others about your TB status? (Why are you hiding your diagnosis? What are you worried about?)
- What has changed after telling them about your diagnosis?
- Have you experienced any changes in the interaction with/behavior of family/friends/work people/community/healthcare workers? (How do they treat you?)
- How do you feel about these changes?

**Stigma experiences:**

- Have you experienced situations where you were treated differently because of your TB status?
- What situations (daily life/work/relationships) have you experienced where you have been treated differently?
- How did you feel in situations where you were treated differently/stigmatized?
- Have you ever felt you were stigmatized because of your TB status?
- In what settings have you experienced stigma? (hospitals/clinics, community/neighbors, home/family, workplace, other settings)
- Did these experiences inhibit you from seeking care/getting an accurate diagnosis/beginning and completing treatment/getting post-treatment follow-up services?
- Have you experienced stigma in hospitals or clinics/from your neighbors in your community where you live/at home/at work that inhibited you from continuing to seek and access TB services?
- Can you tell me more about the TB-related stigma you have experienced?

**Impact:**

- How do these experiences impact/affect your daily life?
- How do they impact/affect your social life?
- How do they impact/affect you economically?

**Effects on health and healthcare:**

- How do these experiences affect your health?
- How do these experiences affect your health-behavior?
- How do they impact your health care?
- Have you thought about other ways of receiving healthcare?
- How do these experiences impact your mental health?
- How do you feel asking for help/seeking help?
- Did you seek healthcare immediately when you noticed your symptoms? (finding out about delay of diagnosis)

**Experiences of others:**

- Do you know of other people with or who have had TB being stigmatized because of their TB status?
- Can you tell me more about the TB-related stigma you have seen or heard of?
- How do you feel about this?

**Coping strategies:**

- How do you handle these experiences and situations? How do you tackle these problems? What do you do in those situations?
- Have you talked to other people about your experiences? Who are you talking to about these experiences?
- What helps you when you feel stressed by these experiences?
- Do you think about these problems?
- How do you handle/react to other people’s behavior/perceptions? (Keep to self, feel alone, avoid others?

**End**

- Is there anything you would like to comment on or that comes to your mind now?
- Would you like to comment on the interview?
- Is there anything left you would like to share?
