# Supplementary figures and images for: Stigmatization and discrimination of female tuberculosis patients in Kyrgyzstan – a phenomenological study
Source: Int J Equity Health. 2025 Jul 1;24:185. doi: 10.1186/s12939-025-02566-4 (PMC12210680; doi:10.1186/s12939-025-02566-4)

**Appendix 3: Coding Guide**

**
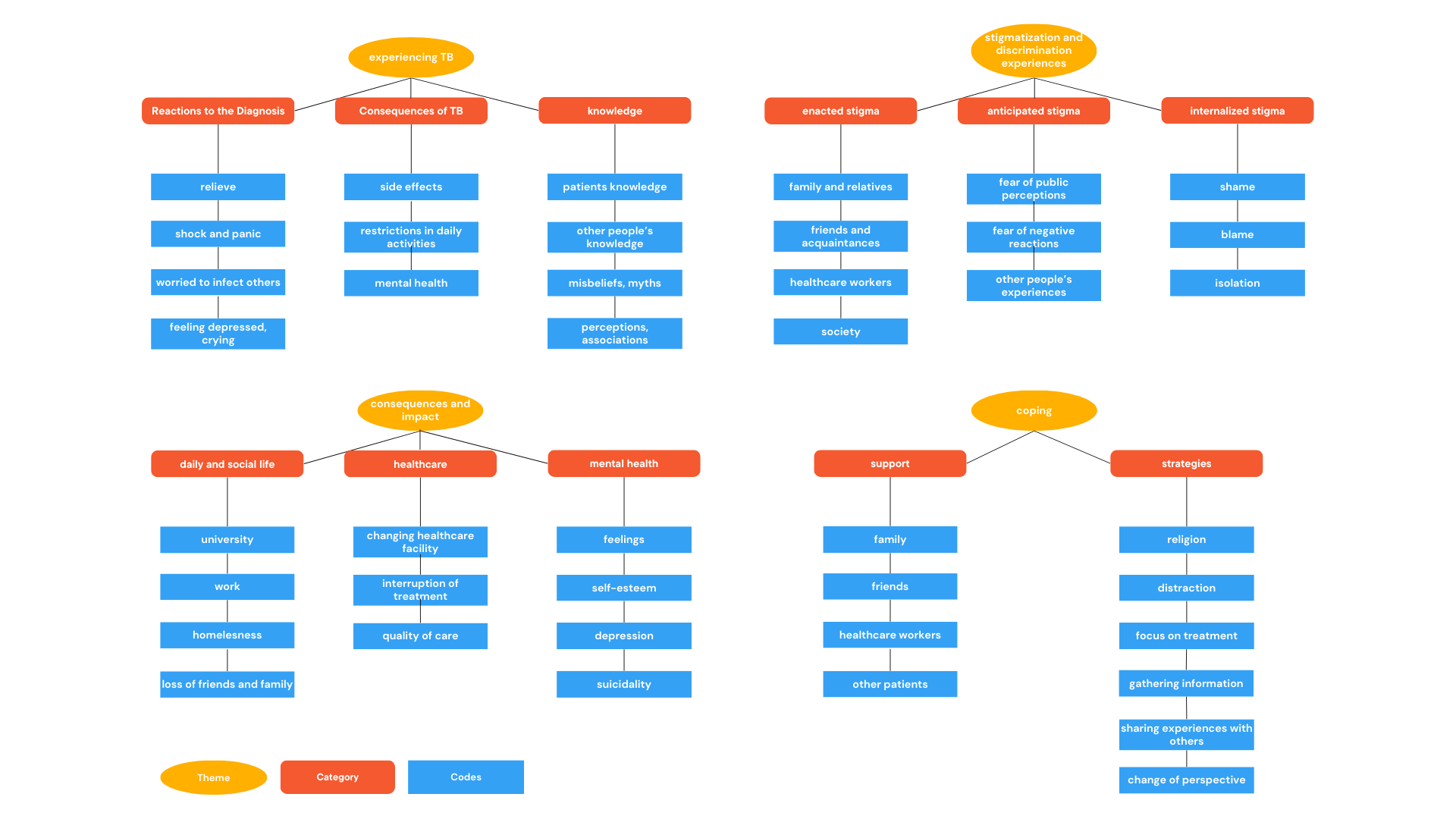
**

Supplement: Supplementary file 3 — Supplementary Material 3. [file 12939_2025_2566_MOESM3_ESM.docx]
