# Supplementary material for: Stigmatization and discrimination of female tuberculosis patients in Kyrgyzstan – a phenomenological study
Source: Int J Equity Health. 2025 Jul 1;24:185. doi: 10.1186/s12939-025-02566-4 (PMC12210680; doi:10.1186/s12939-025-02566-4)
Supplement: Supplementary file 4 — Supplementary Material 4. [file 12939_2025_2566_MOESM4_ESM.docx]

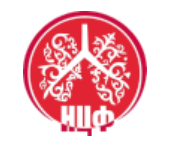

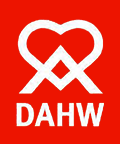
**Appendix 4: Information Sheet**


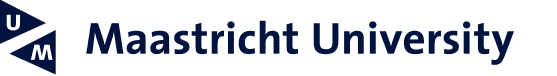


Dear Participant,

I am a master’s student of Global Health at Maastricht University, The Netherlands. I am conducting this interview as part of the data collection for my Master’s Thesis about “The impact of stigmatization and discrimination on female Tuberculosis patients in Kyrgyzstan”. This research will be carried out in collaboration with the National Tuberculosis Program (NTP) Kyrgyzstan and the German Leprosy and Tuberculosis Relief Association (DAHW).

The findings of this study, including some anonymous quotes, will be used for writing my Master Thesis. Additionally, they will be used to write a scientific paper that shall be published in a Global Health journal, like PLOS One or PLOS Global Public Health.

This information sheet is supposed to give you all the information you need to make an informed decision whether you would like to participate in this research or not. Please read carefully, before you sign the informed consent form.

**Title of this research:**

The impact of stigmatization and discrimination on female TB patients in Kyrgyzstan

**What is this research about?**

In Kyrgyzstan many people suffer from TB. There are different factors contributing to that, one of them is stigmatization and discrimination of TB patients. A consequence of the experience of stigmatization and discrimination is delayed healthcare-seeking and discontinuation of treatment. At the same time, stigmatization and discrimination has psychological, economic and social aspects which can decrease the quality of life and mental health. Women often are more affected by stigmatization and discrimination and thus, also its consequences.

**What is the aim of this research?**

The aim of this research is to understand women’s experiences of stigmatization and discrimination after receiving the TB diagnosis. The goal of this study is to create an insight into the consequences of these experiences on an individual person. Thus, the results of this study could be used to create awareness about the impact of TB-stigmatization and discrimination on women to improve their access to healthcare (diagnosis and treatment) and integrate other forms of support (psychological and social) into the treatment. This aims to improve the quality of life and health of affected individuals. Additionally, this research aims to contribute to increased detection and control of TB to end this epidemic one day.

**What is my involvement?**

If you agree to participate in this research, I would like to do an interview with you to ask you some questions about your patient journey. These questions will be about changes in your social, work and daily life after you received the TB diagnosis and told others about it. I would like to hear about your experiences and situations you have been in. I will also ask you how these changes affected your life and your health. Finally, I would like to know how you deal with those changes and situations you find yourself in. You can decide yourself, what you would like to talk about and how many details you would like to reveal. Depending on how much you would like to share, the interviews will take approximately between 30 and 60 minutes.

Before the beginning of the interview, I would like to ask you some questions about your age, religion, ethnicity, marital status, employment and TB diagnosis. This will help me, to put your stories into context. Answering these questions is completely voluntary. If you do not want to answer a question, you can skip it.

**Who will be present during the interview?**

During the interview, you will talk to a female researcher, me, and a female translator. The translator will translate the questions from English to Russian/Kyrgyz and the answers from Russian/Kyrgyz to English to ensure everyone’s understanding. Both, me and the translator, will sign a confidentiality agreement to protect your personal data before the beginning of this study.

**How to participate in this study?**

You can participate in this study if you are a woman over 18 years of age and if you are living in Kyrgyzstan. You need to have a TB diagnosis for which you are currently undergoing treatment. You need to be able to express yourself in English, Russian or Kyrgyz. But most importantly, you need to agree to participate in this study by signing the informed consent form.

**What are my rights?**

The participation in this research is completely voluntary. By signing the informed consent form, you agree to participate in this study. During this study you always have the right to withdraw. You do not need to provide any reason and you will not face any consequences. If you wish to withdraw from the study, your data file will be deleted immediately. Once the data has been analyzed, the anonymized data insights cannot be removed anymore. Although your actual data file will still be deleted.

If you do not feel comfortable in any part of this research (e.g. you do not want to answer a question) you always have the right to skip this part. You can take a break at any point of the interview. If you would like to withdraw from the study, skip a part or take a break, please tell the researcher.

**Will the interviews be recorded?**

If you give your consent, I would like to audio record the interviews and take some written notes during the conversation. Therefore, you need to fill out and sign the informed consent form. If you do not give your consent for the audio recording, unfortunately, you cannot participate in this research.

**How will the data be used?**

The audio recordings of the interviews will be transcribed. The transcriptions and the notes will then be analyzed to find common themes in order to create an understanding of the stigmatization and discrimination experiences of the participants. The results and some anonymous quotes will be used for my Master Thesis. Additionally, they will be used to write a paper that will be published in a Global Health journal. Therefore, the data will be shared with NTP Kyrgyzstan and DAHW who will be part of this research.

**How will the data be stored?**

The data will be safely stored in a password protected folder on a safe server. The data will be stored for ten years after the paper has been published. After that, the data will be destroyed safely. Only authorized members of the research team will have access to the data. These measures are taken to always ensure data protection of the participants.

**Anonymity, Confidentiality, Data Protection**

Throughout the whole process of this research, we will make sure your data is protected. Therefore, all your personal data will be anonymized. We will not use your name; this will be replaced by a pseudonym right from the beginning. Every information that could be traced back to your person, will be anonymized or taken out. By that, we will ensure confidentiality of your responses.

**What are possible risks and their safeguards?**

The topic of stigmatization and discrimination is a very sensitive topic. During the interview you might relive negative emotions or difficult situations you have been in before. This might lead to psychological distress. To avoid this, you can decide yourself what you would like to talk about and how much you would like to share. At any time, you could take a break or end the interview without providing a reason. You do not have to answer a question if you do not want to. You will not be judged. In contrast I would like to encourage you and create a pleasant atmosphere. The interview will take place in a quiet and private environment. You might feel insecure because two strangers will conduct the interview with you. The translator will only be there to translate and avoid misunderstandings. Your responses will not be analyzed or interpreted right away.

**What are my benefits of this research?**

Your contribution in this research could create an understanding of women’s experiences of stigmatization and discrimination after being diagnosed with TB. Thus, the findings of this study could be used to create awareness and consciousness towards your experiences and feelings. Therefore, prevention and intervention strategies could be developed and implemented. This might positively influence social interactions and the quality of your future healthcare. Additionally, in future, social and psychological support could be included in the treatment, which you could make use of, if needed.

**Ethical approval**

To conduct these interviews, ethical approval has been obtained from Maastricht University and from the Ministry of Health in Kyrgyzstan.

**Additional information**

If you like, we can provide you with additional information about the research and its results. We can also provide resources for support (e.g. social or psychological support), if you are interested.

If you have any questions, comments or doubts, you can contact the Ethics Committee at the establishment of the editorial office of the NHRN of the Kyrgyz Republic.

Address: 1 Togolok-Moldo St., building of CRH and MT, 1st floor, 104 office.
