# Supplementary material for: Stigmatization and discrimination of female tuberculosis patients in Kyrgyzstan – a phenomenological study
Source: Int J Equity Health. 2025 Jul 1;24:185. doi: 10.1186/s12939-025-02566-4 (PMC12210680; doi:10.1186/s12939-025-02566-4)
Supplement: Supplementary file 5 — Supplementary Material 5. [file 12939_2025_2566_MOESM5_ESM.docx]

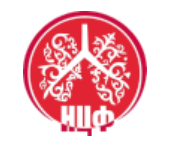

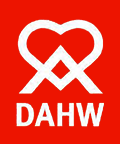

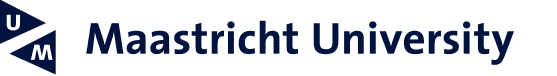
**Appendix 5: informed consent form**

**INFORMED CONSENT FORM**

Dear Participant,

thank you for being part of this research.

Your personal data and everything you say during the interview will be treated with the highest level of care. We ensure data privacy, anonymity and confidentiality. I hereby ask your consent to conduct, record and transcribe the interview and to use the data for scientific purposes, namely my Master Thesis as well as for an article that will be published in a Global Health journal.

If you have any questions, comments or doubts, please do not hesitate to contact me directly.

Rhea Brüggemann, rm.bruggemann@student.maastrichtuniversity.nl

I have been informed about the PURPOSE of this interview YES / NO

I have been informed about potential RISKS of my participation YES / NO

I have had the opportunity to CLARIFY any questions about the interview YES / NO

and my participation in it

I have been informed about my RIGHTS YES / NO

I participate VOLUNTARILY and I understand that I can withdraw from the YES / NO

interview at any point in time without having to provide a reason

I agree to the interview being AUDIO RECORDED YES / NO

I agree to the interviewer taking WRITTEN NOTES during the interview YES / NO

I agree that the interview will be used for SCIENTIFIC purposes YES / NO

I agree that the interview transcription will be SHARED with NTP and DAHW YES / NO

I agree that the interview recording and transcript will be STORED for 10

years after publication YES / NO

Place and Date Place and Date

Name of the participant / signature Name of the researcher/signature
